# End-of-life care for idiopathic pulmonary fibrosis patients with acute exacerbation

**DOI:** 10.1186/s12931-022-02204-5

**Published:** 2022-10-29

**Authors:** Norimichi Akiyama, Tomoyuki Fujisawa, Tatsuya Morita, Takafumi Koyauchi, Yoshinobu Matsuda, Masanori Mori, Mitsunori Miyashita, Ryo Tachikawa, Keisuke Tomii, Hiromi Tomioka, Satoshi Hagimoto, Yasuhiro Kondoh, Yoshikazu Inoue, Takafumi Suda

**Affiliations:** 1 Department of Pulmonary Medicine, Fujieda Municipal General Hospital, 4-1-11 Surugadai, 426-8677 Fujieda, Japan; 2 Second Division, Department of Internal Medicine, Hamamatsu University School of Medicine, 1-20-1 Handayama Higashi-ku, 431-3192 Hamamatsu, Japan; 3 Palliative and Supportive Care Division, Seirei Mikahahara General Hospital, Kita-ku, 3453, 433-8558 Mikatahara, Hamamatsu, Japan; 4 Clinical Research Center, National Hospital Organization Kinki-Chuo Chest Medical Center, 1180 Nagasone-cho, Kita-ku, 591-8555 Sakai, Osaka Japan; 5 Department of Psychosomatic Internal Medicine, Clinical Research Center National Hospital Organization Kinki-Chuo Chest Medical Center, 1180 Nagasone-cho, Kita-ku, 591-8555 Sakai, Osaka Japan; 6 Department of Palliative Nursing, Health Sciences, Tohoku University Graduate School of Medicine, 2-1 Seiryo-machi, Aoba-ku, 980-8575 Sendai, Miyagi Japan; 7 Department of Respiratory Medicine, Kobe City Medical Center General Hospital, 2-1-1 Minatojima-minamimachi, 650-0047 Kobe City, Hyogo Japan; 8 Department of Respiratory Medicine, Kobe City Medical Center West Hospital, 4, 2-chome, Ichibancho, Nagata-ku, 653-0013 Kobe, Japan; 9 Department of Respiratory Medicine and Allergy, Tosei General Hospital, 160 Nishioiwake- cho, 489-8642 Seto, Aichi Japan; 10 Department of Palliative Care Medicine, Tosei General Hospital, 160 Nishioiwake-cho, 489-8642 Seto, Aichi Japan

**Keywords:** Acute exacerbation, End-of-life discussion, Idiopathic pulmonary fibrosis, Palliative care

## Abstract

**Background:**

Acute exacerbation (AE) is a major cause of death in patients with idiopathic pulmonary fibrosis (IPF). AE-IPF patients require optimal palliative care; however, the real-world clinical situations are poorly understood. We aimed to survey the palliative care received by AE-IPF patients, especially with respect to opioid use for dyspnea and the end-of-life discussions (EOLd).

**Methods:**

Self-administered questionnaires were dispatched to 3423 of the certified pulmonary physicians in Japan. They were asked to report a care report form of one patient each with AE-IPF who died very recently about opioid use for dyspnea and EOLd. We further explored the factors associated with the early use of opioids for dyspnea.

**Results:**

Among the 3423 physicians, 1226 (35.8%) returned the questionnaire with the report forms of 539 AE-IPF patients. Of 539 AE-IPF patients, 361 (67.0%) received opioids for dyspnea. Of the 361 patients, 72 (20.0%) received opioids during the initial treatment with an intention of recovery (early use), while 289 (80.0%) did when the recovery was deemed impossible. EOLd was held before the onset of AE in 124 patients (23.0%); however, the majority of patients had EOLd after the admission for AE-IPF. EOLd before the onset of AE was significantly associated with the early use of opioids.

**Conclusion:**

In terminally ill AE-IPF patients, opioids are usually administered when the recovery is deemed impossible, and EOLd are rarely held before the onset of AE. Further studies are warranted on the efficacy of opioids for dyspnea and the appropriate timing of EOLd.

**Supplementary Information:**

The online version contains supplementary material available at 10.1186/s12931-022-02204-5.

## Background

Idiopathic pulmonary fibrosis (IPF) is a progressive disease with a prognosis as worse as that of malignant tumours, such as lung cancer, with poor quality of life (QOL) and severe symptoms such as dyspnea [1–4]. Therefore, palliative care is considered important and has received much attention in recent years [5–9]. In addition, acute exacerbation (AE) is a major cause of death in patients with IPF combined with severe dyspnea and poor prognoses, whose onset is difficult to predict [10–13]. Therefore, AE-IPF is considered one of the major conditions that can be treated with palliative care. However, there have been only a few empirical studies on palliative care for patients with AE-IPF [13]. There are two important aspects of palliative care for AE-IPF: symptomatic relief from dyspnea and decision making at the end-of-life.

IPF patients suffer from a variety of symptoms during the disease course, most frequently dyspnea [5]. Morphine is often used to treat dyspnea in these IPF patients. Although it has been suggested that morphine may be effective and safe for reducing shortness of breath in IPF patients [14], there is currently no evidence on this specific patient population from the prospective randomised trial [15]. In addition, large clinical trials so far that have examined the efficacy of morphine for dyspnea have only included outpatients and there is no report yet on the efficacy of morphine for dyspnea in hospitalised patients with AE-IPF [16]. We, therefore, believe that it is important to clarify how often and when morphine can be administered for dyspnea of IPF patients with AE in a clinical setting.

Another issue with this specific patient population is end-of-life decision making. As in the case of malignant diseases such as lung cancer, IPF patients need to be provided with a forum for advance care planning (ACP) that includes end-of-life issues such as the limits of treatment, respiratory management at the end-of-life and the place to face death [17]. In former reports, several IPF patients reported that they wished to discuss end-of-life issues early in their diagnosis [18, 19]. Recent guidelines recommend that palliative care should be offered to all patients diagnosed with a serious life-limiting illness and not only for IPF patients [20, 21]. However, the ideal timing of ACP cannot be determined uniformly in actual clinical practice. The timing must be considered during interactions with patients and their families while constantly assessing the disease status and the disease progression [17, 22, 23]. In actual clinical practice, end-of-life discussions (EOLd) are often held at the time when the patient’s condition has progressed [6, 7]. In observational studies of IPF patients, the annual incidence of AE is 4 to 20.0 per 100 patient-years, depending on the study populations [10], and AE accounts for 40% of all deaths [11]. AE can occur at any stage of the disease course and is difficult to predict. Thus, it is important to clarify how end-of-life decisions are made for AE-IPF patients.

The primary aim of this study is to describe the palliative care received by AE-IPF patients, especially with respect to opioid use for palliation of dyspnea and decision making at the end-of-life. Furthermore, we aimed to explore the factors associated with the early use of opioids for dyspnea in patients with AE-IPF.

## Methods

This was a nationwide, cross-sectional survey. There were 6846 number of certified pulmonary physicians in Japan. For this study, we randomly identified the half of all the physicians (n = 3426), and questionnaires were distributed to them. These questionnaires were distributed via mail in December 2020, with a reminder sent a month later.

### Subjects and procedure

All pulmonary physicians certified by the Japanese Respiratory Society were recruited for this study. The Japanese Respiratory Society is the only approved organisation that issues a certification of pulmonary physicians. The physicians’ names and respective affiliations were obtained from the website of the said Society. The responses were indicative of the subjects’ consent to participation. No reward was provided for the participation. The responses to the questionnaire were voluntary, and confidentiality was maintained throughout the investigations and subsequent analyses. No identification numbers were linked with the original data.

### Questionnaire and measurement outcomes

The self-administered questionnaire utilised herein was developed based on a review of the literature and our previous research [8, 9, 18–20, 25–27]. The study group (composed of eight pulmonary physicians with expertise in the diagnosis and management of ILD and four palliative care specialists) developed the questionnaire to ensure clarity and comprehensiveness. Face validity was confirmed with a pilot test on 14 pulmonary physicians.

### Care report form of an IPF patient who died from AE

Based on physicians’ recall, the following information was collected on the most recently treated AE-IPF patient who died (1 patient for each physician).　The patient’s age, sex, time from diagnosis to hospitalization, treatment before and after hospitalization and oxygen therapy after hospitalization immediately before death were recorded.

Although we acknowledge the limitation of the reliability and validity of the data about patient status based on the physicians’ recall, we decided to adopt this study design because understanding physician-perceived effectiveness and the appropriateness of the timing of medical interventions could be of value, and the feasibility was high and the limitation was regarded as being acceptable when the aim of the study is hypothesis-gathering.

### Opioid use for dyspnea management

We inquired the participants about whether and when opioids were used for dyspnea of their AE-IPF patient. The potential answers included; (1) used and started from the time of disease-modifying treatment with the intention of recovery (early use), (2) used, but started when the recovery was expected to be impossible (late use) and (3) not used at all. In addition, if opioids were not used, the respondents were asked to provide a specific reason as a free comment. The types of opioids (as-needed oral administration, regular oral administration, or continuous parenteral infusion) were also reported. The physician-perceived effectiveness and physician-perceived timing of opioid use were rated on a 6-point Likert scale (1, not effective at all; 2, very little effect; 3, a little effective; 4, effective; 5, very effective and 6, unevaluable) and a 5-point Likert scale (1, too late; 2, late; 3, appropriate; 4, early; and 5, too late), respectively.

### End-of-life decision making

We inquired the participants to report to whom they talked about end-of-life decision making from the following: (1) talked to patients only, (2) talked to both the patients and their family members, (3) talked to only the family members and (4) did not talk to any of them. End-of-life decision making investigated in this study was defined as discussion about either incurability, estimated prognosis, use of mechanical ventilation when respiratory failure occurs, cardiopulmonary resuscitation, or preferred place of death, according to previous studies [26, 28]. The timing of the talk was also reported from the following: (1) before the onset of AE, (2) on the admission for AE or (3) when recovery was no longer expected. The physician-perceived timing of EOLd was rated on a 5-point Likert scale (1, too late; 2, late; 3, appropriate; 4, early; and 5, too late).

### Background data

As physician backgrounds, information on their age, sex, years of practice, type of hospital, subspeciality and the number of patients with IPF treated in a year, and experience with the national palliative care education programme was recorded by the physicians.

### Statistical analysis

All returned questionnaires were analysed. The frequencies and percentages of eligible participants’ responses to each item were calculated. Fisher’s exact test was performed for comparing the proportions among the groups. The between-group differences were assessed using the Mann-Whitney *U* test.

To explore the factors associated with opioid use, we classified the respondents into two groups based on the timing of opioid administration: (1) patients who received opioids during initial disease-modifying treatment with the intention of recovery (the early use) and (2) patients who received opioids when the recovery was expected to be impossible (the late use). Univariate logistic regression analyses were performed to screen based on the patients’ demographics, that is, age, sex, time from diagnosis to hospitalization, treatment before and after hospitalization and home oxygen therapy before hospitalization. Next, to identify the independent determinants, all factors with p < 0.05 values were identified in univariate analyses and entered into multivariate logistic regression analyses.

All statistical analyses were performed by using EZR (Saitama Medical Center, Jichi Medical University, Saitama, Japan)—a graphical user interface for R (The R Foundation for Statistical Computing, Vienna, Austria) 23—with statistical significance set at p < 0.05 [29]. The approval of the study protocol by an ethics committee was not required according to the national policies in Japan and therefore not obtained. Information that could be used to identify individual patients was not obtained, and the physicians responded spontaneously and anonymously.

## Results

### Participants

**Fig. 1:**
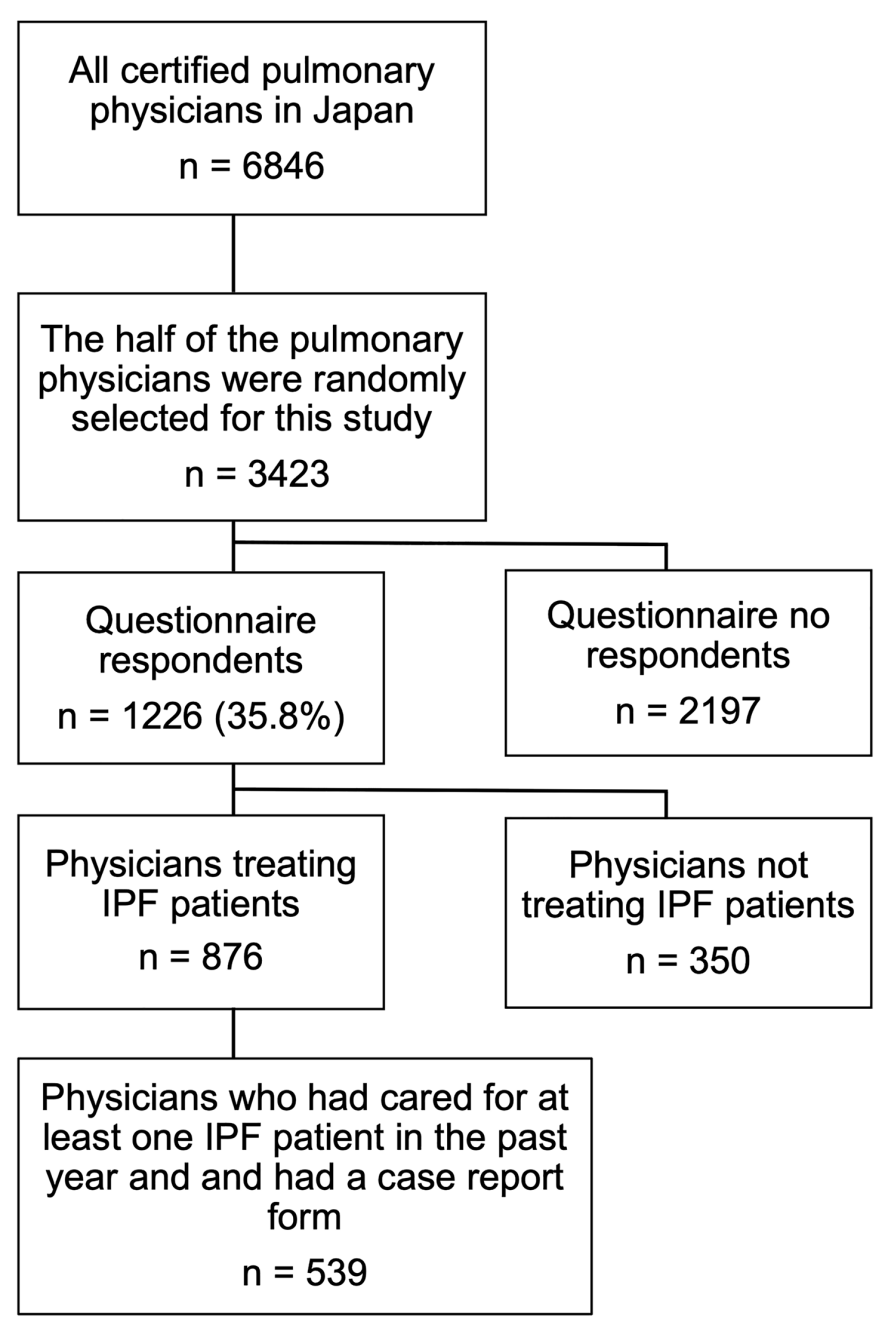
The schematic of the study flow chart IPF: idiopathic pulmonary fibrosis

Among the 3423 eligible participants, 1226 completed the questionnaire (response rate: 35.8%). In the questionnaire respondents, participants who were not engaged in the practice of IPF and had not cared for any IPF patients in the past 1 year or had not listed case reports were excluded from the study (Fig. 1). The participants who had cared for at least one IPF patient in the last year and had a case report form were 539 (44% of the 1226 questionnaire respondents). The characteristics of participants are summarised in Table 1.

**Table 1:** Participant characteristics

	All participants
	(n = 539)
Sex	
Male	447 (82.9)
Female	90 (16.7)
missing	2(0.4)
Age (median, SD)	48 ± 9.9
Years of practice (median, range)	22 (2–60)
Type of hospital	
University hospital	159 (29.5)
Hospital > 500 beds	115 (21.3)
Hospital < 500 beds	239 (44.3)
Medical clinic	25 (4.7)
Other	1 (0.2)
Subspeciality	
Interstitial lung disease	241
Malignant lung tumor	207
Allergic disease	158
COPD	182
Respiratory infectious disease	184
Experience with the national palliative care education program	474 (87.9)
Number of IPF patients cared for within the last year	
1–2	38 (7.0)
3–4	102 (18.9)
5–9	168 (31.2)
> 10	231 (42.9)
Number of IPF patients died in the last year	
1–2	289 (53.6)
3–4	147 (27.3)
5–9	63 (11.7)
> 10	40 (7.4)

S.D., standard deviation; COPD, chronic obstructive pulmonary disease; IPF, idiopathic pulmonary fibrosis

### Characteristics of patients with AE of IPF

The baseline characteristics of patients with AE-IPF are summarised in Table 2. Among the 539 patients, 429 (79.6%) were men. 410 patients (76.1%) had been diagnosed with IPF over a year before hospitalization for AE-IPF, and 126 (23.4%) had done within one year of hospitalization of AE-IPF. A total of 249 (46.2%) patients were treated with an anti-fibrotic agent and 312 (57.9%) patients were treated with home oxygen therapy before hospitalization. Regarding oxygen therapy immediately before death, 179 (33.2%) patients were receiving conventional oxygen therapy, 261 (48.4%) were receiving high-flow nasal cannula and 90 (16.7%) were receiving mechanical ventilation.

**Table 2:** Patient characteristics

	All patients
	(n = 539)
Sex	
Male	429 (79.6)
Female	74 (13.7)
missing	36 (6.7)
Age (median, SD)	70 ± 8.3
Time from diagnosis to hospitalization	
< 3months	42 (7.8)
More than 3 months but less than 6 months	21 (3.9)
More than 6 months but less than one year	63 (11.7)
> one year	410 (76.1)
Missing	3 (0.5)
Treatment before hospitalization	
Antifibrotic agent	249 (46.2)
Corticosteroid	180 (33.4)
Immunosuppressive agent	54 (10.0)
Home oxygen therapy	312 (57.9)
Treatment provided newly after hospitalization	
Corticosteroid	329 (61.0)
Immunosuppressive agent	94 (17.4)
Antifibrotic agent	9 (1.7)
Oxygen therapy after hospitalization	
Conventional oxygen therapy	179 (33.2)
High Flow Nasal Cannula	261 (48.4)
Non-invasive positive pressure ventilation	77 (14.3)
Invasive mechanical ventilation	13 (2.4)
Missing	9 (1.7)

### Opioid use for dyspnea of AE-IPF patients

**Fig. 2:**
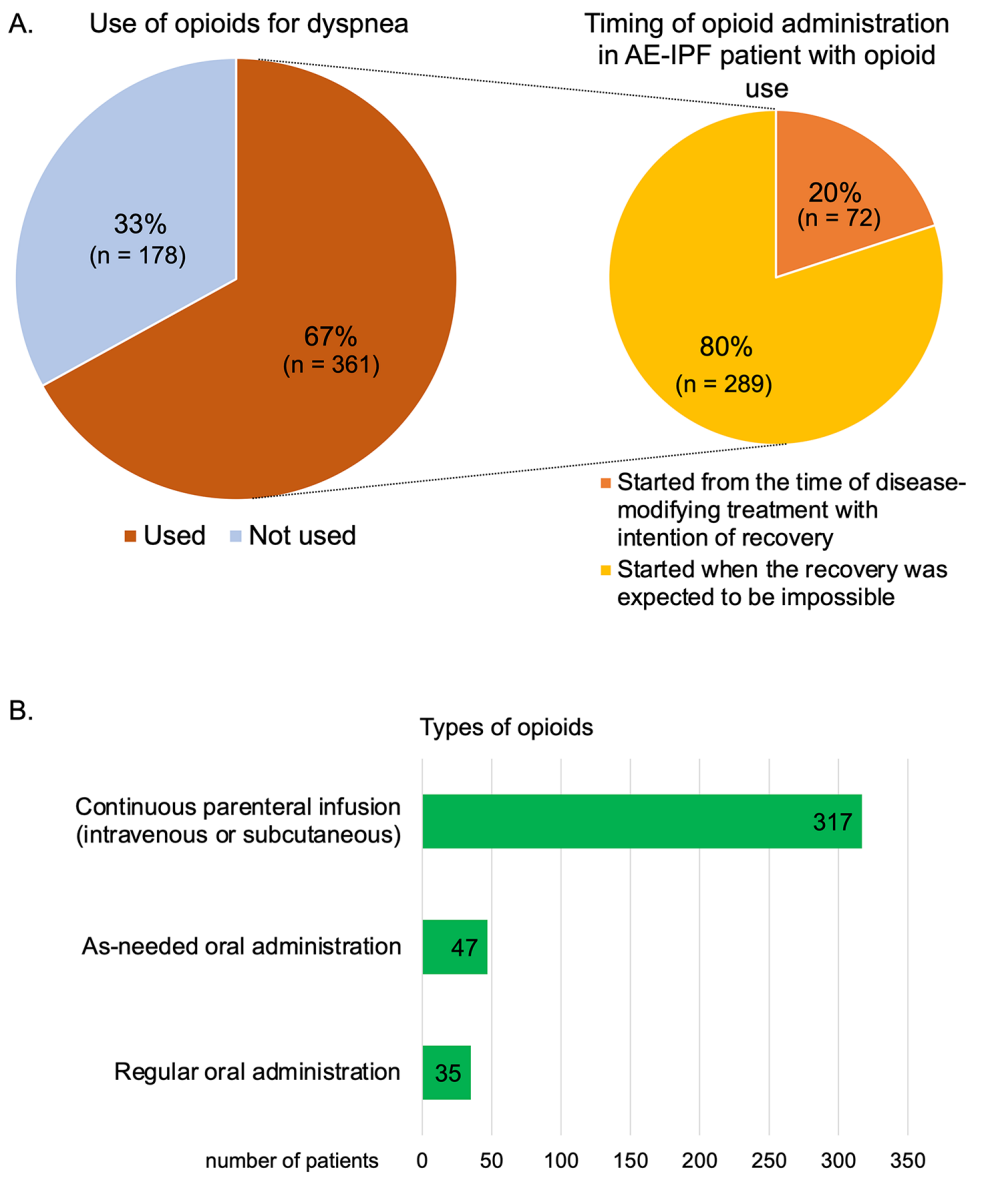
Opioid use for dyspnea in patients with AE-IPF. (A) Proportion of opioid use for dyspnea in patients with AE-IPF (n = 539) and the timing of opioid administration in AE-IPF patient with opioid use (n = 361). (B) Type of opioid used for dyspnea in AE-IPF patients AE: acute exacerbation; IPF: idiopathic pulmonary fibrosis

Of the 539 patients with AE-IPF, 361 (67.0%) patients were administered opioids for dyspnea due to AE and 178 (33.0%) were not (Fig. 2 A). Of the 361 patients who used opioids, only 72 (20.0%) received opioids during the initial disease-modifying treatment with an intention of recovery (early use), while 289 (80.0%) received opioids when the recovery was deemed impossible. The continuous parenteral infusion was the most common type of opioid administration (Fig. 2B). The specific reasons for which opioids were not used for dyspnea are depicted in Supplementary e-Table 1. The three major reasons for not using opioids were as follows: no complaints of dyspnea (n = 34), no time to use opioids due to the rapid progression of the disease (n = 22) and the complications of impaired consciousness (n = 22). Of the 361 patients who used opioids, 217 (60.1%) rated the opioids as effective or very effective for dyspnea and 249 (69.0%) rated the timing of opioid use for dyspnea as appropriate (Table 3). Continuous infusion of parenteral benzodiazepines was performed in 185 (34.3%) patients (data not shown). Comparison of patient backgrounds based on the timing of opioid administration and the early or late use showed no difference in the patient backgrounds before hospitalization between the two groups (Supplementary e-Table 2). There were no significant differences in the administration of corticosteroid and immunosuppressive agent as treatments for AE-IPF between the two groups. The use of high flow nasal cannula as oxygen therapy for AE-IPF was more frequent in the late use group (60.6%) than in the early use group (45.8%). No significant differences were observed in the use of NPPV or invasive mechanical ventilation between the two groups. In the early use group, 59.7% of the participants rated opioids as effective or very effective. In the late-use group, 60.2% of the participants rated the opioids as effective or very effective (Supplementary e-Table 2).

**Table 3:** Physician-perceived effects and timing of opioid use for dyspnea and the timing of EOLd in patients with AE-IPF.

Opioids use n = 361	
Physician- perceived effects of opioids	
Not effective at all	1(0.3)
Very little effect	14 (3.9)
A little effective	118 (32.7)
Effective	169 (46.8)
Very effective	48 (13.3)
Unevaluable	11 (3.0)
Physician-perceived timing when opioids were started	
Too late	8 (2.2)
late	102 (28.3)
Appropriate	249 (69.0)
Early	0
Too early	0
Missing	2 (0.5)
End-of-life discussion n = 536*	
Physician-perceived timing of end-of-life discussions	
Too late	21 (3.9)
late	174 (32.5)
Appropriate	334 (62.3)
Early	1 (0.2)
Too early	0
Missing	6 (1.1)

*Three participants who reported that they did not hold EOLd with either the patients or family members were excluded

### End-of-life decision making of AE-IPF patients

**Fig. 3:**
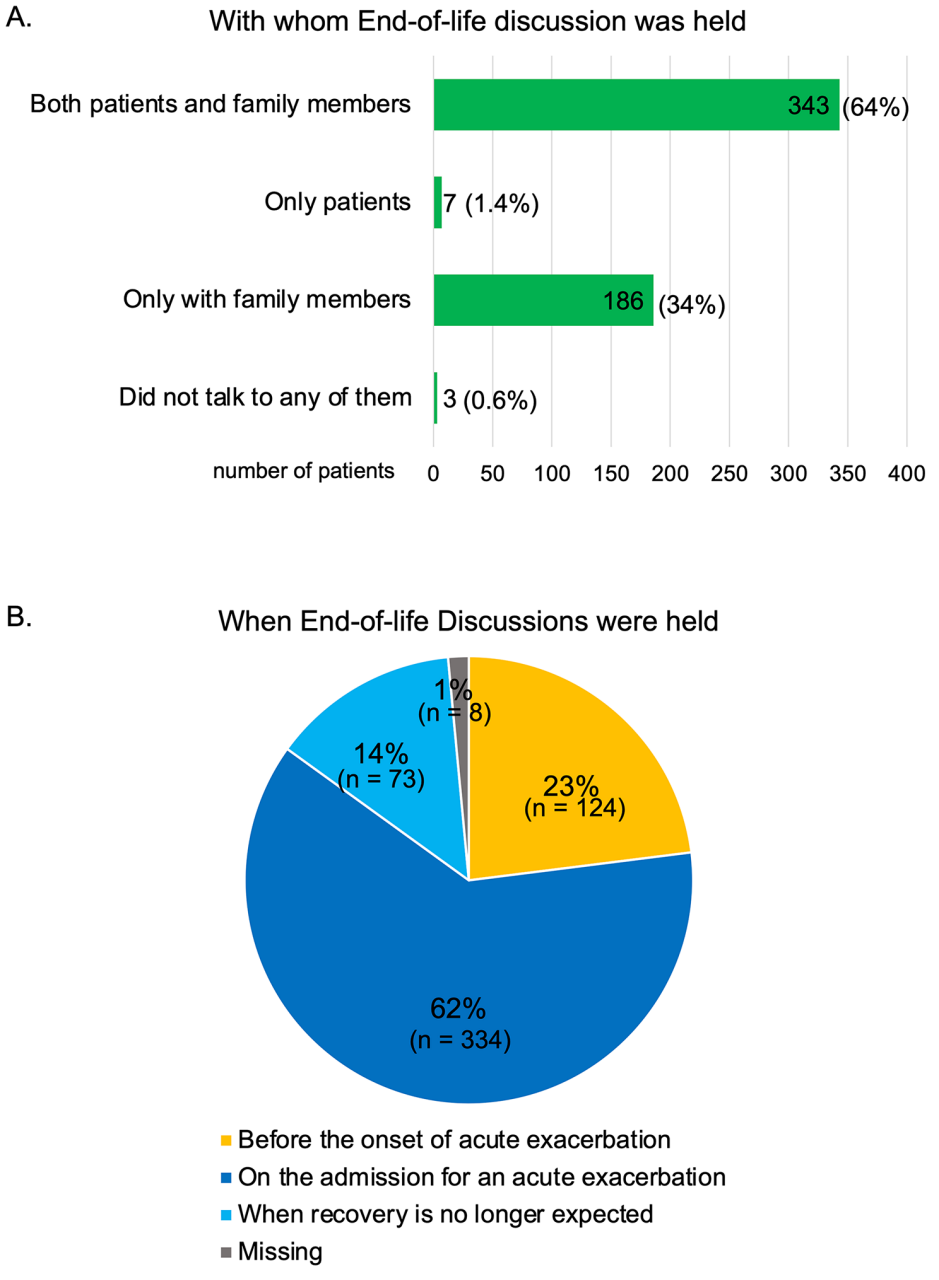
End-of-life discussion in patients with AE-IPF. (A) With whom were the end-of life discussions held. (B) When were the end-of life discussions held. AE: acute exacerbation; IPF: idiopathic pulmonary fibrosis

The majority of the participants (64.0%) reported that they held EOLd with both the IPF patients and the family members (Fig. 3 A). As shown in Fig. 3B, EOLd was held before the onset of AE in 124 patients (23.0%); however, 334 patients (62.0%) had EOLd after the admission for AE-IPF and 73 patients (14.0%) had EOLd when recovery was no longer expected. Of the 536 participants, except for 3, who did not hold EOLd with either the patients or family members, 334 (62.3%) rated the timing of EOLd as being appropriate (Table 3).

Comparison of the patients who had EOLd before hospitalization with those who had EOLd after the hospitalization, the patients with EOLd before hospitalization had a longer period from the time of IPF diagnosis to admission for AE-IPF when compared to those with EOLd after hospitalization (Supplementary e-Table 3). The patients with EOLd before hospitalization received home oxygen therapy more frequently (Supplementary e-Table 3). The appropriate rate of the physician-perceived timing of EOLd was higher in patients with EOLd before hospitalization (81.1%) when compared with those with EOLd after hospitalization (56.5%) (Supplementary e-Table 3).

### Factors associated with the early use of opioids

We next evaluated the factors associated with the early use of opioids for dyspnea in patients with AE-IPF. The results of univariate and multivariate analyses are summarised in Table 4. In the univariate analyses, EOLd before the onset of AE-IPF was significantly associated with the participants’ decisions of early use of opioids (OR 1.89, 95%CI 1.29–2.87, p = 0.002). In contrast, antifibrotic treatment before hospitalization was found to be negatively associated with the participants’ decisions of early use of opioids (OR 0.52, 95%CI 0.38–0.71, p < 0.001). Multivariate analyses revealed that EOLd before the onset of AE-IPF was positively associated with the participants’ decisions of early use of opioids (OR 1.86, 95%CI 1.29–2.87, p < 0.01), and the antifibrotic treatment before hospitalization was negatively correlated with the participants’ decisions of early use of opioids (OR 0.54, 95%CI 0.39–0.75, p < 0.01).

**Table 4:** Factors associated with the early use of opioids: univariate and multivariate analyses

Early use of opioids	Univariate			Multivariate		
Factors	OR	95%CI	p value	OR	95%CI	p value
Age under 70 years	1.24	0.84–1.83	0.29	1.19	0.80–1.76	0.39
Male	0.90	0.57–1.42	0.64	0.96	0.59–1.56	0.87
Hospitalized over one year	1.12	0.75–1.68	0.57	NC	NC	NC
Antifibrotic treatment before hospitalization	0.52	0.38–0.71	< 0.001	0.54	0.39–0.75	< 0.01
Home oxygen therapy before hospitalization	1.33	0.97–1.83	0.08	NC	NC	NC
Corticosteroid before hospitalization	0.87	0.62–1.20	0.39	NC	NC	NC
Immunosuppressive agent before hospitalization	0.96	0.58–1.59	0.86	NC	NC	NC
Antifibrotic treatment after hospitalization	1.28	0.46–3.58	0.64	NC	NC	NC
Corticosteroid after hospitalization	0.84	0.61–1.16	0.29	NC	NC	NC
EOL discussion before the onset of AE-IPF	1.89	1.29–2.87	0.002	1.86	1.29–2.87	< 0.01
EOL discussion with patient	0.40	0.08–2.03	0.27	NC	NC	NC

NC - Not calculated

## Discussion

This nationwide survey of pulmonary physicians revealed the end-of-life care of IPF patients who died of AE. To the best of our best knowledge, no past report has provided detailed information about the end-of-life of IPF patients who died of AE.

The first and most important finding of the present study was to reveal the frequency and timing of opioids use for dyspnea in patients with AE-IPF. In this study, opioids were administered for dyspnea in 67% of the patients with AE-IPF. Not limiting to AE-IPF patients, our previous study reported the end-of-life picture of 177 deceased ILD patients, which included 78 IPF patients and 99 patients who died of AE [30]. In that study, 58.2% of the patients received opioids in the 2 days before death. The rate of opioid use in this study was similar to that in a previous study; these findings confirmed that 60–70% of all IPF patients with fatal complications received opioids at any time of the dying process. In most cases (80%), however, opioids were administered when the recovery was expected to be impossible; in only 20% of the cases, opioids were administered from the time of disease-modifying treatment with the intention of recovery. On the whole, approximately 60% of the participants reported that opioid was effective for dyspnea and that the proportion of participants who believed that the timing of opioid administration was appropriate tended to be higher in the early use group than in the late-use group (79.2% vs. 66.4%; p = 0.096, e-Table 2). These results thus suggest that opioids can be expected to have a certain effect on dyspnea during AE and may be better to start concurrently with the treatment for AE. In the future, a controlled trial would be needed for IPF patients with AE to clarify the best timing for the administration and optimisation of the treatment efficacy.

The second important finding of this study is that only 23% of the IPF patients had EOLd before the onset of AE-IPF. In our previous study, only 13.3% of ILD patients had EOLd before admission, which was similarly low as in the present study [30]. Moreover, 81.1% of the participants who held EOLd before the onset of AE rated the timing of discussions as appropriate when compared with 56.5% of those who held EOLd after hospital admission (p < 0.001, e-Table 3). The results of this study confirmed that pulmonary specialists believe that EOLd should normally be conducted early in the diagnosis of IPF, albeit, in reality, they often took place after the disease had progressed or worsened [28]. The fact that early EOLd is associated with early opioid use suggests that EOLd before the onset of AE-IPF is of great significance to provide appropriate symptom relief for IPF patients. A recent study from Canada on a small population of IPF patients demonstrated that the multidisciplinary care (MDC) model reduced hospital admissions of patients in their last year of life when compared with those not receiving MDC [31]. In this model, ACP discussions were conducted early in the intervention. Several large-scale studies in recent years have demonstrated the effectiveness of early EOLd [32, 33]. Prospective randomized intervention studies including ACP and palliative care from the early stage of diagnosis are underway in IPF patients as well, with results awaited [34].

In the present study, the incidence of acute exacerbations (AE) of IPF within one year after IPF diagnosis was 23.4%, which was relatively higher than that in a former report [10]. As the present study dealt with participants’ most recent experiences of ‘fatal cases of AE- IPF’, the patient’s condition might have been relatively severe at the time of IPF diagnosis, which may be related to a higher incidence of onset of AE within one year after IPF diagnosis. The early onset of AE after diagnosis of IPF may be related to late-use of opioids for dyspnea and less frequent decision-making before the onset of AE in patients with IPF.

In multivariate analyses, antifibrotic treatments before hospitalization are negatively correlated with the decisions of early use of opioids. In terminally ill cancer patients, chemotherapy in the last months of life has been associated with intensive care (e.g., ICU admission, resuscitation) at the end-of-life, which reduces the QOL at the end-of-life and delayed palliative care intervention [35–38]. For patients with IPF, antifibrotic agents are the only treatments that have been proven to decelerate the disease progression [39, 40]. No other effective drugs have been available in the treatment of IPF. In this difficult clinical situation for IPF, physicians may have excessive expectations of the recovery of patients who were treated with antifibrotic agents, which may be associated with the delayed use of opioids in AE-IPF patients treated with antifibrotic agents before their hospitalization.

The strength of this nationwide survey is that the subjects were recruited from pulmonary physicians who treated IPF patients in their daily practice throughout the country, which is highly likely to reflect the actual clinical practice and the number of cases (n = 539) being very large. In addition, since this is the most recent medical experience of participants involved in IPF treatment, it can be treated as the latest data on the death of AE-IPF patients. Despite the strength of the nationwide survey, this study has several limitations. First, this is a questionnaire survey that does not provide detailed patient information based on medical records alone. Second, it may not completely reflect the usual medical practice of the participants as it describes a single case that the participants experienced most recently. Third, the response rate was moderate (35.8%) and the baseline characteristics of non-responders were not available.

## Conclusion

Among the IPF patients who died of AE, the main timing of opioid use for dyspnea was after the physicians had determined that recovery was no longer expected, and EOLd were rarely held before the onset of AE. A high-quality study is highly warranted on the efficacy of opioids for dyspnea and the appropriate timing of EOLd in patients with IPF.

## Electronic supplementary material

Below is the link to the electronic supplementary material.

Supplementary Material 1

## Data Availability

The datasets used and/or analyzed during the current study are available from the corresponding author on reasonable request.

## Abbreviations

**ACP**: Advance Care Planning.
**AE**: Acute Exacerbation.
**EOLd**: End-of-Life discussions.
**IPF**: Idiopathic Pulmonary Fibrosis.
**SD**: Standard Deviation.
