# Supplementary material for: End-of-life care for idiopathic pulmonary fibrosis patients with acute exacerbation
Source: Respir Res. 2022 Oct 29;23:294. doi: 10.1186/s12931-022-02204-5 (PMC9617351; doi:10.1186/s12931-022-02204-5)
Supplement: Supplementary file 1 — Supplementary Material 1 [file 12931_2022_2204_MOESM1_ESM.docx]

**Online Data Supplement**

**e-Table 1.** **Reasons for not using opioids for dyspnea of IPF patients**

| Reasons | n |
| --- | --- |
| No complaints of dyspnoea that would require opioid administration | 34 |
| No time to use opioids due to the rapid progression of the disease | 22 |
| The complications of impaired consciousness | 22 |
| Continuous sedation with midazolam | 12 |
| No insurance coverage for opioids for dyspnoea of IPF patients | 11 |
| The patient did not want to use opioids | 9 |
| Concerns about side effects such as respiratory depression | 6 |
| The family members did not want to use opioids for dyspnoea | 5 |
| Not familiar with opioid use | 5 |
| Invasive mechanical ventilation | 3 |
| The opioids were considered ineffective. | 2 |
| Thought there was a chance of recovery. | 1 |
| Missing | 46 |

**e-Table 2. Patient comparisons (early use opioid or late use opioid).**

|  | Early use | Late use | p.value |
| --- | --- | --- | --- |
| Patients | 72 | 289 |  |
| Age under 70 years | 20 (27.8) | 61 (21.1) | 0.269 |
| Male | 58 (80.6) | 230 (79.6) | 0.938 |
| More than one year from diagnosis to hospitalization | 54 (75.0) | 216 (74.7) | 1 |
| Treatment before hospitalization |  |  |  |
| Antifibrotic agent | 39 (54.2) | 180 (62.3) | 0.226 |
| Corticosteroid | 25 (34.7) | 101 (34.9) | 1 |
| Immunosuppressive agent | 5 (6.9) | 28 (9.7) | 0.648 |
| Home oxygen therapy | 44 (61.1) | 157 (54.3) | 0.354 |
| Treatment provided newly after hospitalization |  |  |  |
| Corticosteroid | 42 (58.3) | 178 (61.6) | 0.686 |
| Immunosuppressive agent | 13 (18.1) | 56 (19.4) | 0.868 |
| Oxygen therapy after hospitalization |  |  |  |
| Conventional oxygen therapy | 30 (41.7) | 94 (32.5) | 0.166 |
| High Flow Nasal Cannula | 33 (45.8) | 175 (60.6) | 0.032 |
| Non-invasive positive pressure ventilation | 12 (16.7) | 37 (12.8) | 0.441 |
| Invasive mechanical ventilation | 3 (4.2) | 2 (0.7) | 0.056 |
| Physician- perceived effects of opioids |  |  |  |
| Effective + Very effective | 43 (59.7) | 174 (60.2) | 1 |
| Physician-perceived timing when opioids were started |  |  |  |
| Appropriate | 57 (79.2) | 192 (66.4) | 0.096 |
|  |  |  |  |

**e-Table 3. Patient comparisons (EOLd before hospitalization or after hospitalization).**

|  | Before hospitalization | After hospitalization | p.value |
| --- | --- | --- | --- |
| Patients* | 127 | 409 |  |
| Age under 70 years | 29 (22.8) | 84 (20.5) | 0.619 |
| Male | 109 (85.8) | 317 (77.5) | 0.129 |
| More than one year from diagnosis to hospitalization | 107 (84.3) | 300 (73.3) | 0.024 |
| Treatment before hospitalization |  |  |  |
| Antifibrotic agent | 66 (52.0) | 229 (56.0) | 0.475 |
| Corticosteroid | 44 (34.6) | 136 (33.3) | 0.830 |
| Immunosuppressive agent | 15 (11.8) | 39 (9.5) | 0.500 |
| Home oxygen therapy | 87 (68.5) | 224 (54.8) | 0.007 |
| Physician-perceived timing of end-of-life discussions |  |  |  |
| Appropriate | 103 (81.1) | 231 (56.5) | <0.001 |

*Three participants who reported that they did not hold EOLd with either patients or family members were excluded.
